# Mechanisms of alveolar type II epithelial cells’ mitochondrial quality control during acute lung injury/acute respiratory distress syndrome: bridging the gap between oxidative stress, inflammation, and fibrosis

**DOI:** 10.3389/fphys.2025.1684729

**Published:** 2025-10-16

**Authors:** Lin Zeng, Jiangtian Yan

**Affiliations:** ^1^ LiShizhen College of Traditional Chinese Medicine, Huanggang Normal University, Huanggang, China; ^2^ Hubei Key Laboratory of Germplasm Improvement and Utilization of Dabie Shan Dao-di Herbs (Huanggang Normal University), Huanggang, China; ^3^ LiShizhen Culture and Industry Research Center of Traditional Chinese Medicine, Huanggang, China

**Keywords:** alveolar type II epithelial cells, mitochondrial quality control, acute lung injury, acute respiratory distress syndrome, oxidative stress, inflammation, fibrosis

## Abstract

Acute lung injury (ALI) and acute respiratory distress syndrome (ARDS) are a group of conditions characterized by acute episodes of pulmonary inflammation and increased pulmonary vascular permeability. These conditions often result in severe morbidity and high mortality rates. Increased alveolar-capillary barrier permeability is a pivotal factor in the pathogenesis of ALI/ARDS, and diffuse alveolar epithelial cell (AEC) death is a salient feature of ALI/ARDS. Alveolar epithelium is composed of alveolar type I epithelial cells (AECI) and alveolar type II epithelial cells (AECII), with AECII playing a more critical role. These cells contain a high density of mitochondria in their cytoplasm, and their function depends on mitochondrial quality control (MQC). Existing reviews either focus solely on the mechanisms of AECs and their relationship to lung injury/fibrosis or broadly explore the role of mitochondrial dynamics in lung diseases. However, neither review comprehensively addresses AECII’s MQC and related molecules and signaling pathways. The objective of this study is to investigate the MQC characteristics of AECII in ALI/ARDS, elucidate their role as a regulatory hub for oxidative stress, inflammation, and fibrosis, summarize progress in related clinical trials, and highlight the need for further research to develop effective therapies.

## 1 Introduction

Acute lung injury (ALI) is a severe respiratory disease with global prevalence, primarily triggered by endogenous and exogenous pathogenic factors (Li J. et al., 2022). The principal clinical manifestations of the condition include uncontrolled oxidative stress, pulmonary oedema, and inflammatory cell infiltration (Fan et al., 2018). ALI has been demonstrated to result in the development of acute respiratory distress syndrome (ARDS) in critical conditions, which is also recognized as a primary cause of mortality in critically ill patients (Li J. et al., 2022; Kraft et al., 2023). As demonstrated in previous studies, the mortality rate for patients with ARDS can reach 34.9%–46.3% (Bellan et al., 2016; Huang et al., 2020). Moreover, even among survivors, a high rate of disability is observed (Moss et al., 2019). Increased permeability of the alveolar-capillary barrier is a key component of the pathogenesis of ALI/ARDS (Wang Z. et al., 2020). Disruption of alveolar epithelial barrier repair has been demonstrated to result in the development of fibrosis in patients with lung injury and to worsen prognosis (Budinger and Sznajder, 2006). To a certain extent, the alveolar epithelial barrier exhibits greater resistance to damage in comparison with the adjacent endothelium (Matthay et al., 1993). As demonstrated in the existing literature, the widespread death of alveolar epithelial cells (AECs) occurs during ALI (Qi et al., 2023; Sha et al., 2024). AECs are considered to be essential parenchymal cells for maintaining the structural and functional integrity of the lungs (Ruaro et al., 2021). The classification of AECs is primarily into two distinct types: alveolar type I epithelial cells (AECI) and alveolar type II epithelial cells (AECII). AECI and AECII are connected via plasma membrane structures (including adherens junctions and tight junctions) (Bhattacharya and Matthay, 2013) to form the alveolar epithelial barrier, a highly compact barrier that restricts solute passage while facilitating carbon dioxide and oxygen diffusion (Matthay et al., 2019a). Of these, the function of AECII is of particular importance.

As is well established, AECII, which possess secretory, proliferative, and innate immune functions, are small cuboidal cells with the anatomical characteristics of actively metabolizing epithelial cells, containing a high density of mitochondria and possessing distinctive apical microvilli (Ruaro et al., 2021). In terms of their functions, they have been observed to secrete surfactants, which have been demonstrated to reduce surface tension, prevent alveolar collapse, and promote efficient gas exchange (Wu and Tang, 2021). Secondly, due to the limited proliferative capacity of AECI, the differentiation and regenerative capacity of AECII is required to restore the barrier function of the alveolar epithelial barrier (Ruaro et al., 2021; Wu and Tang, 2021; Zhang and Liu, 2024; Chong et al., 2023). Research has demonstrated that AECI are particularly vulnerable to damage during the course of ALI/ARDS. Concurrently, AECII undergo cell death when subjected to severe or specific forms of damage. This damage ultimately impairs the ability of AECII to proliferate and differentiate into AECI in a timely manner to repair the extensively damaged alveolar epithelial barrier (Qi et al., 2023). Research has indicated that the lung tissue of animals affected by pneumonia exhibits an increase in the proliferation of AECII. However, these cells have been observed to undergo a loss of mitochondria (Fredenburgh et al., 2015). Finally, it has been demonstrated that AECII may also promote fibrotic responses through the secretion of growth factors and pro-inflammatory molecules following injury (Ruaro et al., 2021). AECII cells that evade cell death adopt a mesenchymal cell fate through a process known as epithelial-mesenchymal transition (EMT). EMT has been demonstrated to maintain the generation of pathologically 'activated’ ATII cells, which in turn amplify the fibrotic response by secreting pro-fibrotic factors, thereby impairing normal post-injury alveolar re-epithelialization (Chilosi et al., 2017; Katzen and Beers, 2020; Paris et al., 2020). Concurrently, studies have demonstrated that tracheal transplantation of human induced-differentiated AECII can terminate and reverse the process of pulmonary fibrosis (Alvarez-Palomo et al., 2020). Therefore, there is a necessity to understand the mechanisms underlying AEC damage during ALI/ARDS and to identify effective intervention strategies. It has been demonstrated that mitochondrial quality control in pulmonary epithelial cells is disrupted during sepsis, leading to mitochondrial dysfunction and the subsequent development of ALI/ARDS (Ning et al., 2022). As demonstrated by the available animal models of ALI, the maintenance of alveolar function by AECII is contingent upon the initiation of MQC (Suliman et al., 2017). The process of MQC is contingent not only on mitochondrial dynamics (fission and fusion) (Shi et al., 2024), but also specifically on mitochondrial autophagy (Zhong et al., 2024).

It is evident that preceding reviews have, in some cases, merely summarized the molecular mechanisms of AECs and their relationship with ALI and fibrosis in isolation. For instance, Katzen provided a comprehensive review of the relationship between AECs quality control and pulmonary fibrosis in 2000 (Katzen and Beers, 2020). Meanwhile, Qi conducted a review in 2023 discussing the interplay between AECII programmed cell death and ALI (Qi et al., 2023). Conversely, there have been reviews that comprehensively summarize the role of mitochondrial dynamics in pulmonary disease. For instance, Sharma (2021) and Li and Pokharel (2024) conducted a comprehensive review of the functions and roles of mitochondrial dynamics in pulmonary disease (Sharma et al., 2021; Pokharel et al., 2024; Li et al., 2024). However, these reviews have not comprehensively addressed the topic of the relationship between MQC in AECII and ALI/ARDS, including the key molecules and signal transduction mechanisms involved. The present study summarizes the MQC process in the dynamic network changes of AECII associated with ALI/ARDS. The integration of signaling molecules and pathways provides further elucidation of the pivotal role of MQC in AECII during ALI/ARDS, and its connections to oxidative stress, inflammation, and fibrosis. In conclusion, the synthesis of evidence from clinical trials demonstrates the mediation of MQC processes in ALI/ARDS. Furthermore, the identification of areas requiring further investigation is pivotal in the development of effective therapeutic strategies for these highly complex pulmonary disorders.

## 2 The important role of alveolar type II epithelial cells in acute lung injury/acute respiratory distress syndrome

### 2.1 Interaction between alveolar type II epithelial cells and major immune cells in ARDS

As is well established, the lungs, as an innate immune organ, contain a multitude of immune cells (including macrophages and neutrophils, among others) (Zeng and Yan, 2025). The surfaces of these immune cells bear numerous pattern recognition receptors (PRRs), which play a regulatory role in pulmonary inflammation through mechanisms such as inflammophagy (Chauhan et al., 2022). Furthermore, the interactions between immune cells and epithelial cells are of paramount importance (Tao et al., 2023). In typical steady-state conditions, the predominant bronchoalveolar cells (BACs) in the lungs are alveolar macrophages (AMs), comprising approximately 90% of the total population (Kumar, 2020). These tissue-resident AMs are formed during the prenatal period and function as sentinel cells to eliminate pathogens (Guilliams et al., 2013). AMs represent a pivotal component of the pulmonary innate immune system (Zeng and Yan, 2025), contributing to inflammatory responses and maintaining homeostasis (Hussell and Bell, 2014). AMs and AECs interact closely, particularly through intercellular interactions involving CD200R, PD-1, and SIRP1α on AMs, and CD200, programmed death-ligand (PD-L)1, and CD47 on AECs (Bissonnette et al., 2020). In pathological conditions, AMs have been shown to promote the repair process of AECs. For instance, TNF-α secreted by AMs has been shown to stimulate granulocyte-macrophage colony-stimulating factor (GM-CSF) production in AECII, thereby promoting AECII proliferation via autocrine signaling (Cakarova et al., 2009). Furthermore, trefoil factor 2 (TFF2) signaling in AMs has been demonstrated to induce Wnt expression, which is essential for AECII proliferation (Hung et al., 2019). In the late phase of ALI, selectively activated AMs secrete anti-inflammatory cytokines that suppress inflammatory responses, promote the proliferation of AECII, and facilitate their differentiation into AECI, thereby aiding alveolar epithelial regeneration and structural remodeling (Tao et al., 2023). Conversely, AECII has also been demonstrated to influence AM activation through the upregulation of glycolysis and oxidative phosphorylation, thereby contributing to lung injury (Li S. et al., 2022). Intrapulmonary or extra thoracic factors attack and disrupt AECs, leading to the release of damage-associated molecular patterns (DAMPs) by AECs. This, in turn, activates AMs and neutrophils, thereby amplifying the inflammatory response and exacerbating ALI (Luo et al., 2023). Research has demonstrated that tissue-resident AMs within barrier organs, serving as the first line of defense against pathogens, can form connexin 43 (Cx43)-containing gap junction channels with epithelial cells (Westphalen et al., 2014). Mice with a specific knockout of AM-expressed Cx43 demonstrated heightened neutrophil infiltration into alveoli and elevated pro-inflammatory cytokine levels in bronchoalveolar lavage fluid (BALF) during Gram-negative bacterial (*Pseudomonas aeruginosa*) pneumonia. In comparison with bacterial pneumonia, the migration of neutrophils to the lungs during sepsis-associated ALI has been shown to exert greater destructive effects on pulmonary tissue (Kumar, 2020). Interactions between AECs (particularly AECII) and AMs have been demonstrated to play a pivotal role in the inflammatory process of pulmonary infection, contributing to the development and resolution of ALI/ARDS.

### 2.2 Alveolar type II epithelial cells and acute lung injury/acute respiratory distress syndrome

**FIGURE 1:**
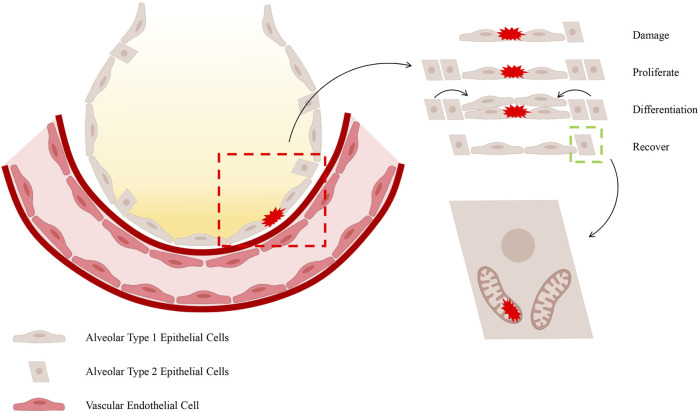
The process of repairing damage to the epithelial barrier. It has been demonstrated that, during the occurrence of ALI/ARDS, AECI are particularly vulnerable to injury. In view of the restricted proliferative capacity of AECI, the differentiation and regenerative capabilities of AECII are imperative for the restoration of the barrier function of the alveolar epithelial barrier. The manner in which AECII provides support to alveolar function is contingent upon the activation of MQC within these cells.

It has been demonstrated that AECII maintains intricate connections with immune cells and that it exerts a direct influence on the integrity of the alveolar membrane. During the pathological development of ALI/ARDS, the disruption of alveolar membrane integrity leads to the formation of protein-rich pulmonary oedema and increased pulmonary fluid (Zeng and Yan, 2025). The alveolar epithelial barrier is subject to disruption by pathogenic factors, including the influx of inflammatory cells and the excessive production of cytokines, chemokines, reactive oxygen species (ROS), and nitrogenous substances (Matthay and Zemans, 2011). Among these, AECI disruption has been shown to compromise the integrity of the alveolar epithelial barrier (Coyne et al., 2003), resulting in the proliferation and differentiation of AECII and the induction of protective epithelial genes that contribute to maintaining tight junctions and restoring membrane integrity (Wray et al., 2009). The existing literature suggests that the MQC process promotes the ability of AECs to eliminate and replace damaged mitochondria, thereby supporting cell survival (Piantadosi and Suliman, 2017). This is followed by the production of mitochondrial autophagy proteins, which are essential for the elimination of damaged mitochondria (Suliman et al., 2017). It has been demonstrated by preceding studies that the proteins which regulate mitochondria appear to be concentrated in AECII. It has been established that these cells are responsible for the production and recycling of surfactant. In addition to this function, they also serve as progenitor cells for AECI, which are essential for alveolar-capillary barrier function and oedema clearance (Suliman et al., 2017). The aforementioned study posits that mitochondrial impairment contributes to alveolar epithelial barrier dysfunction through a series of interconnected processes, including energy depletion, calcium dysregulation, heme homeostasis loss, and cell death activation (Schumacker et al., 2014). Furthermore, due to the substantial surface area and fragility of AECI, in addition to their sensitivity to α-hemolysin lysis and host response-induced cellular damage, the anticipated damage to AECI is extensive. Conversely, cuboidal AECII have been shown to exhibit enhanced resistance to inflammation and oxidative stress (Bhattacharya and Matthay, 2013). It has been demonstrated that surviving AECII are endowed with stem cell functions, which enable them to proliferate and transdifferentiate into AECI, thus helping to restore alveolar-capillary barrier function (Barkauskas et al., 2013) (Figure 1). However, AECII is only able to fulfil this function by first repairing cellular damage, eliminating mitochondria with irreversible damage, and replacing them with healthy mitochondria to support cellular survival (Suliman et al., 2017).

## 3 The role of mitochondrial quality control processes in alveolar type II epithelial cells in acute lung injury/acute respiratory distress syndrome

As demonstrated by the extent animal models of ALI, the provision of AECII support for alveolar function is contingent on mitochondrial biogenesis (Athale et al., 2012) and the activation of the MQC programme (Suliman et al., 2017). Of these, the MQC process appears to be of greater significance (Jiang Y. et al., 2022), primarily divided into two components: mitochondrial dynamics (Whitley et al., 2019) and mitochondrial autophagy (Chang et al., 2015). The MQC process has been demonstrated to enhance the ability of AECs to eliminate and replace damaged mitochondria, thereby supporting cellular survival (Piantadosi and Suliman, 2017). In addition to laboratory-based research, some researchers conducted a histopathological case-control autopsy study, which revealed severe mitochondrial oxidative damage in AECII of patients who died from ARDS, along with impaired clearance of damaged mitochondria (Kraft et al., 2023). This underscores the necessity of studying the MQC process in AECII for the prevention and treatment of ALI/ARDS. The present review also summarizes the corresponding basic research, including cell experiments (Ning et al., 2022; Ballweg et al., 2014; Liu W. et al., 2019; Luo et al., 2019; Kim et al., 2020; Zhuang et al., 2021; Zhang et al., 2021; Zhao et al., 2021; Chen et al., 2022; Tian et al., 2022; Liu et al., 2021; Guan et al., 2021; Kia et al., 2021; Jiang J. et al., 2022; Zhu et al., 2022a; Yang et al., 2022; Lin Q. et al., 2022; Liu et al., 2022; Wang et al., 2023; Xuefei et al., 2023; Dong et al., 2023; Zhang et al., 2023; Li N. et al., 2023; Liu et al., 2023; Song et al., 2023; Zhou et al., 2023; Han et al., 2023; Li C. et al., 2023; Chu et al., 2024; Su et al., 2024; Zhou et al., 2024; Zhu et al., 2024; Yu et al., 2024; Lian et al., 2024; Han et al., 2025; Liu et al., 2025; Peng et al., 2025; Gu et al., 2025; Tomatis et al., 2025) (Table 1) and animal experiments (Ning et al., 2022; Suliman et al., 2017; Liu W. et al., 2019; Luo et al., 2019; Zhuang et al., 2021; Liu et al., 2021; Zhu et al., 2022a; Li N. et al., 2023; Liu et al., 2023; Song et al., 2023; Han et al., 2023; Zhu et al., 2024; Han et al., 2025; Liu et al., 2025; Ping et al., 2025; Wang et al., 2024; Meng et al., 2025) (Table 2). The two tables presented herein provide substantial experimental evidence for the association between the MQC process and ALI/ARDS in AECII. Specifically, both AECII and various animal models of ALI/ARDS induced by multiple mechanisms exhibit oxidative stress, inflammation, and fibrosis, all of which are closely linked to MQC. Direct modulation of the MQC process, or indirect regulation via signaling molecules/pathways, has been shown to effectively suppress the onset and progression of oxidative stress, inflammation, and fibrosis.

**TABLE 1:** Related study on mitochondrial quality control mechanism of AECII.

Cell lines	Stimulus/Inducing factor	Cellular response	Mitochondrial quality control	Intervention/Therapeutic factor	Signaling molecules/pathways
MLE-12, pmATII. (Ballweg et al., 2014)	CSE	Oxidative stress, and inflammation	Mitochondrial dynamics (fusion↑, and fission―), Mitophagy―	None	None
pmATII. (Liu et al., 2019a)	LPS	Inflammation	Mitophagy↓	TFEB overexpression	TFEB
A549. (Luo et al., 2019)	LPS	The results in this section are illustrated by animal experiments	Mitophagy↑	Mdivi-1	None
MLE-12. (Kim et al., 2020)	Oxidant	Oxidative stress	Mitophagy↓	reOGG1	reOGG1
MLE-12. (Zhuang et al., 2021)	LPS	Oxidative stress, and inflammation	Mitophagy↑	MCTR3	ALX/PINK1 signaling pathway
MLE-12. (Zhang et al., 2021)	CSE	Oxidative stress	Mitophagy↓	RAPA, MitoQ, and SRT1720	Sirt1
A549. (Zhao et al., 2021)	LPS	Oxidative stress	Mitochondrial dynamics (fusion↓), Mitophagy↑	Oxyberberine	Parkin/PINK1 Pathway
A549. (Chen et al., 2022)	TNF-α	Oxidative stress, and inflammation	Mitochondrial dynamics (fission↑), Mitophagy↑	Vit. D	AKT and NF-κB pathways
HPAEpiCs. (Tian et al., 2022)	LPS, and TGF-β1	Oxidative stress, inflammation, and EMT (fibrosis)	Mitophagy↓	Tβ4	None
pmATII. (Liu et al., 2021)	LPS	Inflammation	Mitophagy↓	PGC-1α overexpression	TFEB
A549. (Guan et al., 2021)	CSE	Oxidative stress	Mitochondrial dynamics (fusion↓, and fission↑), Mitophagy↓	STS	Sirt1 pathway
A549. (Kia et al., 2021)	CEES	Oxidative stress, and inflammation	Mitochondrial dynamics (fusion↓)	Curcumin	None
RLE-6TN. (Jiang et al., 2022b)	Hyperoxia-induced	None	Mitochondrial dynamics (fusion↓, and fission↑)	None	ERK1/2-dependent
pmATII. (Zhu et al., 2022a)	LPS	Oxidative stress	Mitochondrial dynamics (fusion↓)	NR4A1 deletion	NR4A1
A549. (Yang et al., 2022)	LPS	Oxidative stress, and inflammation	Mitochondrial dynamics (fusion↓, and fission↑)	Kinsenoside	AMPK/Nrf2 Pathway
A549. (Lin et al., 2022a)	CSE, and PMS	Inflammation	Mitophagy↑	NEAT1 siRNA	NEAT1/PINK1 pathway
A549. (Liu et al., 2022)	PQ	Oxidative stress	Mitochondrial dynamics (fusion↓)	MitoQ	None
A549. (Wang et al., 2023)	CSE	Oxidative stress, and inflammation	Mitochondrial dynamics (fission↑), Mitophagy↓	UA	None
A549, pmATII. (Ning et al., 2022)	LPS	Oxidative stress, and inflammation	Mitochondrial dynamics (fusion↓, and fission↑), Mitophagy↑	Melatonin	Sirt3/SOD2 pathway
RLE-6TN. (Xuefei et al., 2023)	Hyperoxia-induced	Oxidative stress	Mitophagy↑	None	None
MLE-12. (Dong et al., 2023)	CSE	Oxidative stress	Mitochondrial dynamics (fusion↓, and fission↑)	PRDX2 overexpression, and NAC.	PRDX2
MLE-12. (Zhang et al., 2023)	PQ	Oxidative stress, inflammation, and EMT (fibrosis)	Mitochondrial dynamics (fission↑), Mitophagy―	Mdivi-1, and NAC.	TLR9/NF-κB signaling pathway
pmATII. (Li et al., 2023a)	LPS	Oxidative stress	Mitochondrial dynamics (fusion↓, and fission↑), Mitophagy↑	Transfected with siHDAC3	HDAC3/FOXO1/ROCK1 axis
A549. (Liu et al., 2023)	PM_2.5_	Oxidative stress, and inflammation	Mitochondrial dynamics (fusion↓, and fission↑), Mitophagy↑	Mdivi-1, BGP-15	None
pmATII. (Song et al., 2023)	LPS	Oxidative stress	Mitochondrial dynamics (fusion↓, and fission↑)	BEZ	PPAR-γ-dependent manner
MLE-12. (Zhou et al., 2023)	S1P	Fibrosis	Mitochondrial dynamics (fusion↓, and fission↑)	JTE-013	RHOA/YAP pathway
A549. (Han et al., 2023)	Human recombinant gVPLA2	None	Mitochondrial dynamics (fusion↓, and fission↓)	None	cPLA2/PGE2 signaling pathway
A549. (Li et al., 2023b)	CSE	Oxidative stress	Mitochondrial dynamics (fusion↓, and fission↑), Mitophagy↑	Leflunomide, BGP-15, Mfn2 overexpression, OPA1 overexpression	None
MLE-12. (Chu et al., 2024)	BLM	Fibrosis	Mitophagy↓	Tetrandrine	PINK1-Parkin signaling pathway
MLE-12. (Su et al., 2024)	BLM	Fibrosis	Mitochondrial dynamics (fusion↓, and fission↑)	YAP1 overexpression	YAP1-Prdx3 axis
A549. (Zhou et al., 2024)	LPS	Oxidative stress, and inflammation	Mitophagy↓	HSPB8 overexpression	HSPB8
A549. (Zhu et al., 2024)	LPS	The results in this section are illustrated by animal experiments	Mitophagy↓	BCAP31 overexpression	PINK1/Parkin pathway
MLE-12. (Yu et al., 2024)	TNF-α	Oxidative stress	Mitophagy↑	LGS	None
A549. (Lian et al., 2024)	None	Oxidative stress	Mitochondrial dynamics (fusion↓, and fission↑), Mitophagy↑	PTPN21 overexpression	PTPN21
MLE-12. (Han et al., 2025)	LPS	Oxidative stress, and inflammation	Mitophagy↓	TBC1D15 overexpression	TBC1D15
RLE-6TN. (Liu et al., 2025)	Hyperoxia-induced	Oxidative stress, and inflammation	Mitochondrial dynamics (fusion↓), Mitophagy↓	oe-Mfn1, si-gadd7 and Rapa	LncRNA gadd7, LSD1/H3K9me3 pathway
RLE-6TN. (Peng et al., 2025)	PQ	EMT (fibrosis)	Mitophagy↑	GBE	p38 MAPK pathway
RLE-6TN. (Gu et al., 2025)	PS-MPs	Oxidative stress, and inflammation	Mitochondrial dynamics (fission↓), Mitophagy↑	None	None
A549. (Tomatis et al., 2025)	YFV infection	Oxidative stress	Mitochondrial dynamics (fusion↑, and fission↓), Mitophagy↑	None	None

**TABLE 2:** Related study on AECII’ mitochondrial quality control mechanism of ALI/ARDS.

Induction method	Pulmonary response	Mitochondrial quality control	Intervention/Therapeutic factor	Signaling pathways
*S. aureus* pneumonia , C57BL/6J mice. (Suliman et al., 2017)	Oxidative stress, and inflammation	Mitochondrial dynamics (fusion↑, and fission↑), Mitophagy↑	None	None
LPS, Sprague-Dawley rats. (Liu et al., 2019a)	Inflammation	Mitophagy↓	TFEB overexpression	TFEB
LPS, Sprague-Dawley rats. (Luo et al., 2019)	Oxidative stress, and inflammation	Mitophagy↑	Mdivi-1	None
LPS, C57BL/6 mice. (Zhuang et al., 2021)	Oxidative stress, and inflammation	Mitophagy↑	MCTR3	ALX/PINK1 signaling pathway
LPS, Sprague-Dawley (SD) rats. (Liu et al., 2021)	Inflammation	Mitophagy↓	PGC-1α overexpression	TFEB.
LPS, WT mice and NR4A1^−/−^ mice. (Zhu et al., 2022a)	Oxidative stress, and inflammation	The results in this section are illustrated by cell experiments	NR4A1^−/−^	NR4A1
LPS, C57BL/6 mice, Sirt3 knockout mice. (Ning et al., 2022)	Oxidative stress, and inflammation	Mitochondrial dynamics (fusion↓, and fission↑), Mitophagy↑	Melatonin	Sirt3/SOD2 pathway
LPS, C57BL/6 mice, Sftpc-CreERT2^+^-HDAC3^flox/flox^ (HDAC3 CKO) mice, Sftpc-CreERT2^-^-HDAC3^flox/flox^ (HDAC3-C) mice. (Li et al., 2023a)	Oxidative stress, and inflammation	Mitochondrial dynamics (fusion↓, and fission↑), Mitophagy↑	HDAC3 CKO	HDAC3/FOXO1/ROCK1 axis
PM_2.5_, C57/BL6 mice. (Liu et al., 2023)	Inflammation	Mitochondrial dynamics (fusion↓, and fission↑)	Mdivi-1, BGP-15	None
LPS, C57BL/6 mice. (Song et al., 2023)	Oxidative stress, and inflammation	Mitochondrial dynamics (fusion↓, and fission↑), Mitophagy↑	BEZ	PPAR-γ-dependent manner
HTV, C57BL/6J mice. (Han et al., 2023)	Inflammation	Mitochondrial dynamics (fusion↓, and fission↓)	Clodronate liposomes and varespladib pre-treatment 2 × 10^6^ Bone marrow-derived macrophages for 24 h	cPLA2/PGE2 signaling pathway
CLP, BALB/c mice. (Ping et al., 2025)	Oxidative stress, and inflammation	Mitophagy↑	Gyp-XLIX	Sirt1/Nrf2 signaling pathway
EHS, Sprague-Dawley (SD) rats. (Wang et al., 2024)	Oxidative stress, and inflammation	Mitophagy↓	Parkin overexpression	PINK1/Parkin pathway
LPS, WT mice, AECII BCAP31-deficient mice (BCAP31^CKO^), Sftpc-BCAP31 transgenic mice (BCAP31^TG^). (Zhu et al., 2024)	Oxidative stress, and inflammation	Mitophagy↓	BCAP31^CKO^	PINK1/Parkin pathway, mROS/ROS/NF-κB pathway
LPS, C57BL/6J mice. (Han et al., 2025)	Oxidative stress, and inflammation	The results in this section are illustrated by cell experiments	TBC1D15 overexpression	TBC1D15
EHS, Sprague-Dawley (SD) rats. (Meng et al., 2025)	Oxidative stress, and inflammation	Mitophagy↓	Parkin overexpression	PINK1/Parkin-mediated mitophagy dysfunction
Hyperoxia-induced, Sprague-Dawley (SD) rats. (Liu et al., 2025)	Oxidative stress, and inflammation	The results in this section are illustrated by cell experiments	shRNA-gadd7 adenovirus vector treatment	LncRNA gadd7, LSD1/H3K9me3 pathway

### 3.1 The role of mitochondria in acute lung injury/acute respiratory distress syndrome

Mitochondria are dynamic, multifunctional organelles that produce adenosine triphosphate (ATP) and numerous biosynthetic intermediates through oxidative phosphorylation (OXPHOS) in response to the cell’s bioenergetic and biosynthetic demands. It is important to note that, in contrast to nuclear organelles, mitochondria are the only non-nuclear organelles that possess their own genome. This genome encodes a total of 13 polypeptides that function as OXPHOS subunits and components of the respiratory chain. In addition to these polypeptides, the mitochondrial genome encodes two ribosomal RNAs and 22 transfer RNAs. These latter components are essential for the process of polypeptide translation in human and mouse mitochondria. Furthermore, mitochondria have been identified as the primary source of endogenous ROS (Fang et al., 2020). Furthermore, mitochondria have been demonstrated to facilitate the temporal storage of calcium ions (Ca^2+^), a process that is imperative for the maintenance of cellular calcium homeostasis (Rizzuto et al., 1993; Baughman et al., 2011). It is imperative to note that other significant metabolic reactions that occur in mitochondria include the synthesis of steroid hormones and porphyrins, the urea cycle, lipid metabolism, and the interconversion of amino acids.

During the process of consuming oxygen to produce cellular ATP, the mitochondrial electron transport chain of the OXPHOS complex transfers single electrons to oxygen, forming ROS through complexes I and III, primarily superoxide and hydrogen peroxide. Mitochondria possess an antioxidant defense system that functions to detoxify and minimize ROS. This mitochondrial redox buffering capacity is precisely controlled to avoid mitochondrial dysfunction and cell death. However, under various pathophysiological conditions caused by hypoxia, ischaemia/reperfusion injury, chemical stress, drug therapy, genetic defects, or metabolic fluctuations, mitochondrial reactive oxygen species (mtROS) levels increase (Cho and Kleeberger, 2020). Impaired mitochondrial function has been demonstrated to exert a detrimental effect on cellular metabolism, resulting in the production of deleterious ROS (Sabouny and Shutt, 2020; Cheung et al., 2024). The production of mtROS in cells can occur in two different ways. Firstly, it can be produced directly within the electron transport chain as a byproduct of oxidative phosphorylation. Secondly, it can be induced by nearby ROS, serving as both a source and target of ROS (Kattoor et al., 2017). A number of studies have demonstrated that mitochondrial autophagy is inhibited in patients with sepsis (Liu et al., 2024). Furthermore, it has been established that calcium overload and increased ROS levels resulting from mitochondrial dysfunction lead to increased cell death (McClintock et al., 2022). As the condition worsens, increased mtROS and mediator production in mitochondria activate downstream cellular processes, including inflammation, fibrosis, and cell death (Suryadevara et al., 2019). Increased cellular fragmentation has been demonstrated to inhibit ATP production, and to result in the leakage of dysfunctional mitochondrial DNA (mtDNA) into the cytoplasm (Nakada et al., 2009). This, in turn, has been shown to lead to increased ROS and inflammation. Furthermore, a specific association between increased mitochondrial fragmentation and enhanced production of cytokines and chemokines has been reported (Tiku et al., 2020).

**FIGURE 2:**
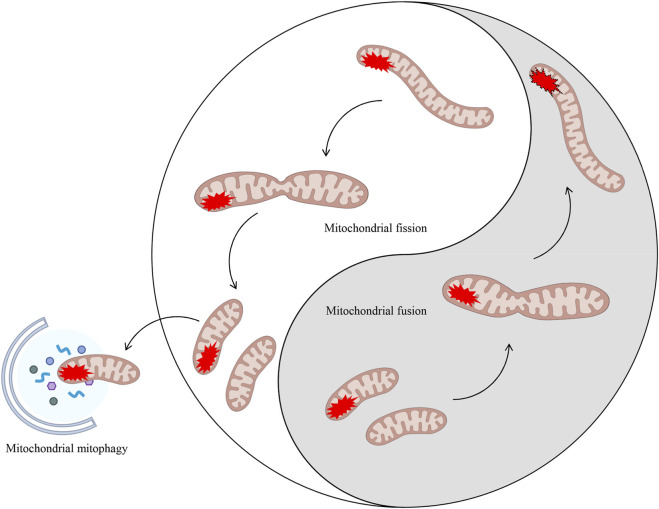
Mitochondrial dynamics (fission/fusion) and mitochondrial autophagy both eliminate damaged mitochondria. MQC encompasses mitochondrial dynamics (fission and fusion) and mitochondrial autophagy. The processes of fission and fusion within mitochondrial dynamics are analogous to the ancient Chinese philosophical concept of Taiji. These processes dynamically regulate damaged mitochondria within the cell through two entirely opposing yet unified processes, thereby maintaining intracellular homeostasis. In cases where mitochondrial damage is irreparable, a process known as mitochondrial autophagy may be initiated in order to eliminate the damaged components.

The normal functioning of mitochondria in healthy cells is contingent on two processes: bioenergetics (ATP production) and biogenesis (the increase in mitochondrial mass by *de novo* generation) (Cho and Kleeberger, 2020; Popov, 2020). In the event of impaired mitochondrial function, the MQC process becomes imperative for the elimination and replacement of damaged mitochondria (Lemasters, 2005; Adebayo et al., 2021). The MQC is comprised of two principal components: mitochondrial dynamics and mitochondrial autophagy (lysosome-dependent selective degradation of defective mitochondria). The term 'mitochondrial dynamics’ refers to the dynamic nature of mitochondria, which are organelles responsible for maintaining the stability of mitochondrial morphology and function. This is achieved through continuous processes of fission, defined as the binary division of mitochondria, and fusion, characterized as the mixing of contents within a mitochondrial population (Whitley et al., 2019). Consequently, mitochondrial health is pivotal to the cellular determination of apoptosis and necrotic cell death programmes, thus rendering quality control a pivotal regulatory factor for cellular survival (Jiang Y. et al., 2022). It is evident that several mechanisms of mitochondrial quality control have evolved, with mitochondrial fission/fusion dynamics and mitochondrial autophagy being of particular significance (Ashrafi and Schwarz, 2013) (Figure 2).

### 3.2 The processes of mitochondrial dynamics and mitochondrial autophagy

#### 3.2.1 Mitochondrial dynamics (fusion/fission)

From a narrative perspective, the term 'mitochondrial dynamics’ encompasses the processes of mitochondrial fusion and fission (Jiang Y. et al., 2022). Mitochondria are highly dynamic organelles capable of altering their size, shape, and location within mere seconds (Scott and Youle, 2010). The number and morphology of mitochondria are known to vary depending on cell type and demand (Wai and Langer, 2016). It is therefore the case that the balance between mitochondrial fusion and fission is a key regulator of mitochondrial distribution, morphology, and function. The process of mitochondrial fusion and fission is facilitated by the action of large multi-domain guanosine triphosphate hydrolases (GTPases) associated with dynamin. These GTPases function by self-assembling to remodel different membranes within the cell (Hoppins et al., 2007).

**FIGURE 3:**
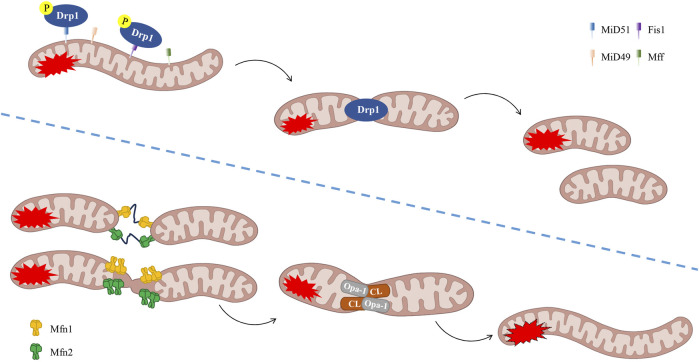
The process of mitochondrial dynamics (fission/fusion). Phosphorylation of Drp1 has been demonstrated to promote fission. It is evident that Fis1, Mff, MiD49 and MiD51 play a pivotal role in the process of fission. Mitochondrial fusion is defined as the process of interconnectedness between two or more mitochondrial networks, resulting in the formation of a unified structural entity. It is imperative to note that the following fusion regulators are of particular significance: OPA-1, CL and Mfn1/2. CL, lipid cardiolipin, Drp1, dynamin-related protein 1, Fis1, fission 1, Mff, mitochondrial fission factor, Mfn1, mitofusin 1, MiD49, mitochondrial dynamics proteins of 49 kDa, MiD51, mitochondrial dynamics proteins of 51 kDa, OPA1, optic atrophy 1.

The process of mitochondrial fusion has been demonstrated to facilitate the mixing of mitochondrial matrix proteins and outer and inner mitochondrial membrane proteins, thereby promoting material exchange and ATP production (Farmer et al., 2018). During mild to moderate cellular stress, the process of fusion between damaged and healthy mitochondria may occur, thereby preventing cellular damage. The process of mitochondrial fusion is primarily associated with two distinct classes of GTPases: mitofusin 1 (Mfn1) and Mfn2 act on the outer mitochondrial membranes (OMM), whilst optic atrophy 1 (OPA1) acts on the inner mitochondrial membranes (IMM). Following the promotion of OMM fusion by Mfn1 polymerisation, OPA1 spiral assembly initiates the process of IMM fusion (Yapa et al., 2021) (Figure 3). The long OPA1 (L-OPA1) has been observed to bind to IMM via trans-lipid cardiolipin (CL). L-OPA1 has been demonstrated to promote mitochondrial fusion, and under conditions of mitochondrial stress, it undergoes proteolytic cleavage to form short OPA1 (S-OPA1) (Lin J. et al., 2022). Subsequently, S-OPA1 was found to add to the L-OPA1-CL complex, fusing with the mitochondrial membrane (Ban et al., 2017).

On the other hand, mitochondrial fission cleaves mitochondrial tubules and produces shorter, more mobile, and isolated mitochondria. These can migrate to other regions of the cell or fuse with other mitochondrial tubules. In the process of mitochondrial fission, two small mitochondria undergo rupture at the mitochondrial-endoplasmic reticulum contact region. The primary mediators of this mechanism are dynamin-related protein 1 (Drp1), mitochondrial fission factor (Mff), mitochondrial dynamics proteins of 49 kDa (MiD49), and mitochondrial dynamics proteins of 51 kDa (MiD51), which are important regulatory factors in mitochondrial fission. It has been established that Drp1 is the primary fission regulator and that it belongs to the dynein family. The process of calcium-regulated phosphatases removing phosphate from Drp1 (which is typically found in the cytoplasm) has been shown to attract it to the mitochondrial surface (Giacomello et al., 2020). Concurrently, it adheres to the mitochondrial surface via bridging proteins (including Fis1, Mff, MiD49, and MiD51) to participate in fission (Quiles and Gustafsson, 2022). In summary, the process of actin aggregation at the mitochondria-endoplasmic reticulum contact sites (MERCs) is followed by the preparation of the mitochondria to undergo contraction. Concurrently, a Drp1 ring is assembled at the site of contraction (Korobova et al., 2013) (Figure 3). Research has demonstrated that signal transducers and activators of transcription 2 (Stat2) play a pivotal role in the regulation of mitochondrial fission homeostasis. Stat2 has been demonstrated to promote the phosphorylation of Drp1 at the S616 site, thereby facilitating the translocation of Drp1 to the mitochondria, thus preparing them for fission (Yu et al., 2020). Furthermore, Toll-like receptors (TLRs) represent a significant class of receptors within the innate immune system, playing a pivotal role in inflammatory responses during ALI/ARDS (Zeng and Yan, 2025). Research has demonstrated that TLR4 inhibitors have the capacity to impede the translocation of Drp1 to the mitochondria. This process has been shown to result in a reduction of Drp1 phosphorylation and myeloid differentiation primary response protein 88 (MyD88) expression in cells. Consequently, this regulatory mechanism functions to control mitochondrial fission through the inhibition of the TLR4/Drp1 pathway. This, in turn, has the effect of modulating the inflammatory response (Zhou et al., 2019). The fusion of remaining healthy mitochondria after fission has been demonstrated to promote mitochondrial oxidative phosphorylation and to allow mtDNA to redistribute between damaged and healthy mitochondria (Huang et al., 2023). This process is believed to contribute to the maintenance of mitochondrial homeostasis.

Mitochondrial dynamics represent a significant adaptive response to acute cellular stress in numerous cell types, including endothelial cells. However, under prolonged stress, initial damage can lead to overcorrection, thereby impairing the cell’s ability to effectively regulate its mitochondria (Eisner et al., 2018), and consequently causing numerous harmful effects. Consequently, the processes of mitochondrial fusion and fission collaborate to facilitate the repair of damaged mitochondria. This is achieved by the separation of damaged components through fission and the exchange of materials between healthy mitochondria through fusion. This collaborative process ensures the maintenance of mitochondrial quality (Ni et al., 2015). Research has demonstrated that nuclear factor erythroid 2-related factor 2 (Nrf2) can exert a protective effect by regulating mitochondrial dynamics. Nrf2 can be activated by excessive ROS, thereby upregulating Mfn2 expression and downregulating Drp1 expression, thus restoring the balance of mitochondrial dynamics, promoting the recovery of healthy mitochondria, and exerting a protective effect on the body (Chen, 2022). Furthermore, studies have demonstrated that Nrf2 has the capacity to inhibit not only pyroptosis, but also ferroptosis, by targeting the regulation of mitochondrial dynamics, as facilitated by Drp1 and Mfn2 (Sun et al., 2023). Moreover, the process of fission facilitates the separation of damaged mitochondrial fragments from the mitochondrial network, thereby enabling their selective removal from the cell via mitochondrial autophagy (Jiang Y. et al., 2022). Consequently, drugs that modulate mitochondrial fusion and fission dynamics, in addition to mitochondrial autophagy, have the capacity to inhibit the accumulation of damaged mitochondria. This, in turn, promotes cell survival and serves as a means to improve sepsis treatment.

#### 3.2.2 Mitochondrial autophagy

When damaged, mitochondria separate from the mitochondrial network through a process of fission, and subsequently, mitochondrial autophagy degrades them to maintain a healthy mitochondrial pool (Cho and Kleeberger, 2020). Following the complete cleavage of the mitochondria into two parts, the autophagy system selectively targets damaged mitochondria and degrades them through lysosomal fusion to maintain mitochondrial quality control. This process is known as mitochondrial autophagy (Lemasters, 2005).

**FIGURE 4:**
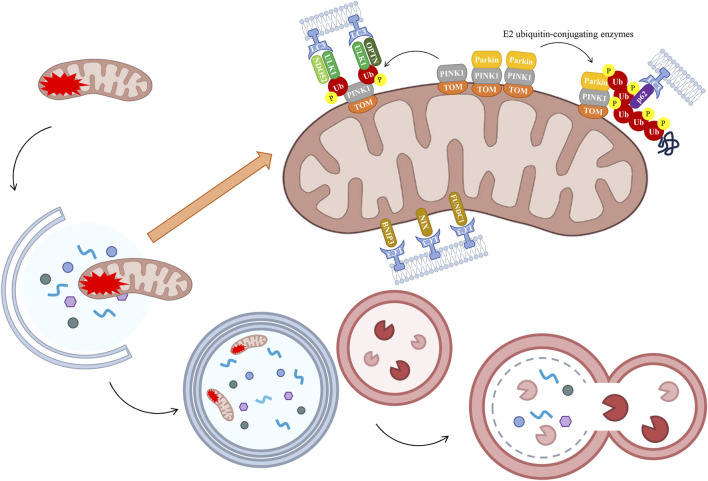
The process of mitochondrial autophagy. Under normal conditions, PINK is transported to the IMM, where it undergoes degradation. In instances of severe damage, the process of mitochondrial fission occurs, resulting in the segregation of damaged segments. The resulting damaged mitochondrial fragments exhibit reduced mitochondrial membrane potential, thereby inhibiting PINK1 translocation to the IMM. PINK1 accumulates on the OMM, where its kinase activity is activated by autophosphorylation, which in turn triggers the recruitment of Parkin. Parkin has been shown to ubiquitinate OMM proteins, interacting with LC3 to induce phagosome assembly and subsequent formation of the autophagosome. It is noteworthy that alternative receptors have been identified which circumvent the PINK1/Parkin pathway-mediated ubiquitination process, thereby facilitating a direct initiation of mitochondrial autophagy through the action of proteins such as BNIP3, NIX, and FUNDC1. The fusion of these two types of vesicles leads to the process of autophagy, or “self-eating”, as the damaged mitochondrial fragments are cleared. Healthy mitochondrial fragments have been shown to retain their functional integrity and to fuse with the wider mitochondrial network. BNIP3, BCL2 interacting protein 3, FUNDC1, FUN14 domain containing 1, LC3, light chain 3, NIX, NIP3-like protein X, OPTN, optic nerve phosphodiesterase, PINK1, PTEN-induced putative kinase 1, TOM, translocase of the outer mitochondrial membrane, Ub, ubiquitin, ULK1, UNC-51-like kinase 1.

The PTEN-induced putative kinase 1 (PINK1)/Parkin signaling pathway has been the subject of extensive research as a mechanism leading to mitochondrial autophagy. Mitochondrial impairment has been demonstrated to induce a decline in membrane potential, thereby impeding the entry of PINK1 into the inner mitochondrial membrane and resulting in its accumulation on the outer mitochondrial membrane (Georgakopoulos et al., 2017). PINK1 accumulates on the OMM, forming a complex with the translocase of the outer mitochondrial membrane (TOM). This process promotes phosphorylation of PINK1 at Ser65, which in turn activates Parkin recruitment and ubiquitin ligase activity. Consequently, Parkin facilitates the ubiquitination of diverse mitochondrial proteins through a synergistic interaction with E2 ubiquitin-conjugating enzymes, culminating in the envelopment of damaged mitochondria by phosphorylated ubiquitin chains (Quinn et al., 2020). The autophagy adaptor protein p62 is recruited to mitochondria, a process which is crucial for final clearance. p62 is a substrate for autophagy that has been the focus of a great deal of study. During the process of autophagy, p62 functions as a link between microtubule-associated protein 1A/1B-light chain 3 (LC3) and polyubiquitinated proteins. This process enables the selective isolation of these proteins into autophagosomes, thereby facilitating the selective recruitment of ubiquitinated substrates for degradation via autophagy. These substrates are subsequently degraded by lysosomal proteases (Bartlett et al., 2017). In the presence of the LC3 complex, the process of autophagy (self-eating) involves the engulfment of damaged mitochondria by autophagosomes, leading to the subsequent fusion of these vesicles with lysosomes for degradation (Ng et al., 2021) (Figure 4). Defects in mitochondrial dynamics and autophagy have been demonstrated to increase levels of mtROS and activate Nrf2. Activation of Nrf2 has been demonstrated to increase the expression of PINK1 and p62, promote the function of mitochondrial autophagy, and clear damaged mitochondria (Chen, 2022). Furthermore, enhanced mitochondrial autophagy, as facilitated by PINK1/Parkin, has been shown to inhibit NOD-like receptor protein 3 (NLRP3) inflammasome assembly, thereby alleviating interleukin (IL)-1β- and IL-18-induced joint inflammation. However, treatment with the mitochondrial autophagy inhibitor 3-MA has been shown to reverse the inhibitory effect on NLRP3 inflammasome activation (Fan et al., 2021). Further studies have shown that sirtuins 1 and 3 (Sirt1/3) can activate the PINK1/Parkin axis, thereby interfering with and inhibiting pyroptosis. Conversely, Sirt1 inhibitors or Parkin silencing have been shown to reverse the expression of NLRP3 inflammasome-associated proteins and promote pyroptosis (Guo et al., 2021). Abnormal expression of the Sirt1/Sirt3 axis has been demonstrated to inhibit the PINK1/Parkin signaling pathway. This results in impaired mitochondrial ubiquitination and disruption to the intracellular mitochondrial autophagy balance. This results in the death of cells due to excessive accumulation of Fe^2+^. This process leads to the occurrence of lipid peroxidation and an accumulation of ROS (Liao et al., 2023).

Furthermore, PINK1 has been observed to trigger mitochondrial autophagy in a manner that is independent of Parkin recruitment. In circumstances where mitochondrial function has been impaired, the protein PINK1, located on the OMM, has been observed to recruit two other proteins, nuclear dot protein 52 (NDP52) and optic nerve phosphodiesterase (OPTN), to the mitochondria. This process has been shown to activate a key initiator of autophagy, known as UNC-51-like kinase 1 (ULK1), thereby inducing mitochondrial autophagy (Lazarou et al., 2015). In addition, a significant number of receptors located on the OMM have the capacity to bind to LC3. These receptors have been shown to circumvent the ubiquitinisation process facilitated by the PINK1/Parkin pathway, thereby enabling direct initiation of mitochondrial autophagy. This process involves the participation of proteins such as BCL2 interacting protein 3 (BNIP3), NIP3-like protein X (NIX), and FUN14 domain containing 1 (FUNDC1) (Wilhelm et al., 2022) (Figure 4). When Nrf2 mediates the action of antioxidant enzymes, BNIP3/NIX-mediated mitochondrial autophagy exhibits high sensitivity to inhibiting ferroptosis, thereby restoring intracellular mitochondrial ROS levels and reducing the occurrence of ferroptosis (Yamashita et al., 2024). Research has demonstrated that FUNDC1-mediated mitochondrial autophagy and pyroptosis are interconnected processes. Activation of FUNDC1 has been demonstrated to promote mitochondrial autophagy and preserve mitochondrial function by activating the adenosine 5′-monophosphate (AMP)-activated protein kinase (AMPK)-ULK1 axis (Zhao et al., 2024). This process has been shown to reduce ROS production, thereby inhibiting NLRP3 inflammasome recruitment-induced pyroptosis.

### 3.3 The mitochondrial quality control of alveolar type II epithelial cells bridges the gap between oxidative stress, inflammation, and fibrosis

In patients suffering from ALI/ARDS, ROS have been shown to have numerous potential sources, including inflammatory cells (neutrophils, monocytes, and macrophages) and parenchymal cells (endothelial cells and epithelial cells, fibroblasts, and muscle cells) (Liu and Chen, 2017). As demonstrated in seminal studies undertaken hitherto, it has been established that mitochondria are the primary contributors to the phenomenon of cellular oxidative stress (Zhou et al., 2017; Zhu et al., 2022b). This phenomenon can be attributed to the heightened sensitivity of mtDNA to oxidants in comparison to nuclear DNA. The impairment of mtDNA, consequent to oxidant damage, gives rise to a series of deleterious consequences, including impaired electron transport chain function and loss of mitochondrial membrane potential. These phenomena ultimately result in mitochondrial kinetic defects (Liu and Chen, 2017). Damaged and dysfunctional mitochondria, in turn, produce greater amounts of ROS, leading to a positive feedback loop that propagates further oxidant-driven damage (Budinger et al., 2011; Kellner et al., 2017; Puri and Naura, 2020; Wang M. et al., 2020). Inflammation and oxidative alveolar damage are hallmarks of ALI/ARDS and result in damage to epithelial cells and subcellular components (e.g., nuclear DNA and mitochondria) (Kellner et al., 2017; Chow et al., 2003). In essence, inflammation plays a pivotal role in the initial pathogenesis of pulmonary fibrosis (PF). Dysfunction of AECs and the subsequent inflammatory response have been shown to be pivotal in initiating the fibrotic process, which in turn leads to extracellular matrix deposition and lung tissue remodeling (O'Dwyer et al., 2019). Consequently, the correction of MQC within AECII serves to address the interconnection between oxidative stress, inflammation, and fibrosis, a subject that will be further explored in the subsequent sections.

#### 3.3.1 Oxidative stress

During ALI, an excess of ROS in the mitochondria can result in ATP deficiency, loss of mtDNA integrity, and cytoplasmic oxidative burst, ultimately leading to cell death. This finding suggests that preserving the balance of mitochondrial dynamics is essential for the proper functioning of lung epithelial cells (Yang et al., 2022; Shi et al., 2021). The generation of mtROS within cells can occur in two distinct ways. Firstly, it can be produced as a byproduct of oxidative phosphorylation within the electron transport chain. Secondly, it can be induced by nearby ROS, thereby serving as both a source and a target of ROS (Kattoor et al., 2017). Elevated ROS levels have also been demonstrated to promote mtDNA oxidation, which in turn leads to an elevated mutation rate in mtDNA (Kaarniranta et al., 2019). It has been established that the ultimate consequence of mtDNA dysregulation is metabolic dysfunction and inflammation (Yuan et al., 2022). The preservation of mtDNA integrity in AECII has been shown to play a crucial role in the mitigation of oxidative stress-induced damage in murine lung epithelial (MLE)-12 cells (Kim et al., 2020).

Mitochondrial dynamics have been demonstrated to be crucial for the exchange of metabolites and mtDNA. Increased fragmentation within cells has been demonstrated to inhibit ATP production, with the concomitant leakage of dysfunctional mtDNA into the cytoplasm resulting in further increased ROS and inflammation (Nakada et al., 2009). Further studies have demonstrated that, in the context of lung injury, ROS can function as second messengers, engaging with tissue and immune cells to amplify immune responses and thereby intensify disease severity (Zhang et al., 2018). A pivotal transcription factor, Nrf2, modulates the synthesis of antioxidant proteins that eliminate ROS (Liu Q. et al., 2019). As a transcription activator of antioxidant response element (ARE) genes, it remains inactive when bound to Kelch-like ECH-associated protein 1 (KEAP1) in the cytoplasm. Upon activation, Nrf2 undergoes phosphorylation, subsequently translocates to the nucleus, and binds to ARE, thereby regulating the increased expression of antioxidant genes, including superoxide dismutase (SOD), glutathione peroxidase (GSH-Px), and heme oxygenase-1 (HO-1) (Zhao et al., 2017; Shaw and Chattopadhyay, 2020). The loss or genetic mutations of Nrf2 have been demonstrated to result in oxidative stress-induced mitochondrial dysfunction and metabolic disorders (Ludtmann et al., 2014; Kovac et al., 2015). Furthermore, substantial evidence suggests a direct association between Nrf2 and mitochondrial antioxidant defense, bioenergetic processes, mitochondrial autophagy, and dynamics (Piantadosi et al., 2008; Mitsuishi et al., 2012; Hayes and Dinkova-Kostova, 2014; Cho et al., 2019). Research has demonstrated that cigarette smoke extract (CSE) induces oxidative stress in A549 cells. However, following treatment of cells with leflunomide (Mfn2 promoter) and BGP15 (OPA1 promoter), as well as Mfn2 overexpression (OE) and OPA1 OE, the CSE-induced oxidative stress was significantly alleviated (Li C. et al., 2023). A substantial body of fundamental literature has demonstrated that MQC is closely associated with oxidative stress-induced damage in AECII, triggered by multiple factors (Tables 1, 2). Key protein molecules and signaling pathways include: the antioxidant enzyme peroxiredoxin-2 (PRDX2) (Dong et al., 2023) and peroxisome proliferator-activated receptor-γ (PPAR-γ) (Song et al., 2023), which mediate mitochondrial dynamics; Sirt1 (Zhang et al., 2021; Guan et al., 2021), and the Parkin/PINK1 pathway (Zhao et al., 2021) have been demonstrated to mediate mitochondrial autophagy; The AKT and nuclear factor kappa-B (NF-κB) pathways (Chen et al., 2022), non-receptor protein tyrosine phosphatase 21 (PTPN21) (Lian et al., 2024) has been shown to mediate MQC. Consequently, the mitigating effects of oxidative stress in AECII, induced by multiple factors, can be achieved.

#### 3.3.2 Inflammation

ROS has been identified as a significant contributor to oxidative stress, and the excessive accumulation of ROS has been demonstrated to result in cellular damage and cell death (Zeng and Yan, 2025; Min et al., 2021). This results in the release of a large amount of cellular contents into the extracellular matrix, triggering a strong inflammatory response and promoting a feedback loop between cellular damage and inflammation, thereby exacerbating tissue damage (Quan et al., 2024). Specifically, a large number of immune cells aggregate at the injury site, initiating a series of inflammatory signal transduction pathways and releasing a large amount of pro-inflammatory cytokines. Disruption of the barrier function of alveolar epithelium and vascular endothelium has been demonstrated to result in an increase in the permeability of the alveolar capillary membrane. This results in the accumulation of protein-rich oedematose fluid in the pulmonary interstitial, which ultimately leads to pulmonary oedema and tissue damage (Matthay et al., 2019a; Mowery et al., 2020). The key transcription factor Nrf2 mentioned earlier has also been observed to regulate the NLRP3 inflammasome, mitogen-activated protein kinase (MAPK), and NF-κB signaling pathways to prevent inflammation and oxidative stress (Ng et al., 2014). Research has demonstrated that exposure to PM2.5 can trigger a cascade of oxidative stress and inflammatory responses, resulting from the disruption of MQC in AECII. However, following the administration of mitochondrial division inhibitor-1 (Mdivi-1) (a Drp1 inhibitor) or BGP-15 (an OPA1 activator) to cells and animals, or following the administration of Drp1 knockdown (KD) and OPA1 OE to cells, has been shown to effectively mitigate the onset and progression of oxidative stress and inflammation (Liu et al., 2023). Furthermore, a substantial corpus of fundamental research literature suggests that oxidative stress damage and severe inflammatory responses frequently co-occur in AECII induced by multiple factors and are closely associated with the MQC process (Tables 1, 2). The key protein molecules and signaling pathways involved include: It has been demonstrated that nuclear receptor subfamily 4 group a member 1 (NR4A1) (Zhu et al., 2022a), AMPK/Nrf2 pathway (Yang et al., 2022), and cytoplasmic phospholipase A2 (cPLA2)/prostaglandin E2 (PGE2) signaling pathway (Han et al., 2023) can mediate mitochondrial dynamics. Transcription factor EB (TFEB) (Liu W. et al., 2019), peroxisome proliferator-activated receptor gamma (PPARγ) coactivator 1α (PGC-1α) (Liu et al., 2021), nuclear enriched abundant transcript 1 (NEAT1) (Lin Q. et al., 2022), heat shock protein B8 (HSPB8) (Zhou et al., 2024), PINK1/Parkin pathway (Zhu et al., 2024; Wang et al., 2024; Meng et al., 2025), TBC domain family member 15 (TBC1D15) (Han et al., 2025) have been identified as mediators of mitochondrial autophagy. The AKT and NF-κB pathways (Chen et al., 2022), Sirt3/SOD 2 pathway (Ning et al., 2022), histone deacetylase 3 (HDAC3) (Li N. et al., 2023), PPAR-γ (Song et al., 2023), and long non-coding RNA (lncRNA) growth-arrested DNA damage-inducible gene 7 (gadd7) through the lysine-specific demethylase 1 (LSD1)/H3K9me3 pathway (Liu et al., 2025) have been shown to mediate MQC. It is evident that, in view of the above key signaling molecules and signaling pathways, oxidative stress damage and intense inflammatory responses induced by multiple factors can be alleviated by regulating MQC in AECII.

#### 3.3.3 Fibrosis

Mitochondria have been demonstrated to play a critical role in the fate of AECII and pulmonary fibrosis (Larson-Casey et al., 2020). Dysfunctional mitochondria have been shown to produce excessive ROS, which in turn activate stress pathways that promote the fibrotic transformation of AECII (Kim et al., 2016; Zhang et al., 2025). The preservation of mtDNA integrity in AECII has been demonstrated to play a pivotal role in the mitigation of fibrotic processes induced by oxidative stress (Kim et al., 2020). The absence of Mfn1 and Mfn2 in mouse AECII has been demonstrated to result in the onset of disease and pulmonary fibrosis. Furthermore, the absence of Mfn1 and Mfn2 in AECII, in conjunction with the inhibition of lipid synthesis via fatty acid synthase deficiency, has been shown to exacerbate bleomycin (BLM)-induced PF (Chung et al., 2019). Furthermore, studies have demonstrated that lipid deficiency can impair progenitor cell renewal capacity in AECII during the process of aging and idiopathic pulmonary fibrosis (IPF) (Liang et al., 2024). A further study established that the expression of fatty acid synthase in AECII serves to alleviate BLM-induced PF by restoring mitochondrial dysfunction in mice (Shin et al., 2023). Thymosin β4 (Tβ4) has been demonstrated to possess antioxidant, anti-inflammatory, and anti-fibrotic properties. A study found that intraperitoneal adeno-associated virus-Tβ4 (AAV-Tβ4) can inhibit oxidative stress and inflammatory responses by promoting mitochondrial autophagy, ultimately attenuating transforming growth factor (TGF)-β1-induced EMT in HPAEpiC (Tian et al., 2022). The use of Mdivi-1 (a Drp1 inhibitor) has been demonstrated to effectively inhibit paraquat (PQ)-induced oxidative stress damage and EMT, as well as TLR9/NF-κB (Zhang et al., 2023). Furthermore, a substantial corpus of foundational literature research suggests a close relationship between fibrosis in AECII induced by multiple factors and the MQC process (Tables 1, 2). The key protein molecules and signaling pathways involved are as follows: the Ras homolog gene family, member A (RHOA)/yes-associated protein (YAP) pathway (Zhou et al., 2023), the YAP1-Peroxiredoxin 3 (Prdx3) axis (Su et al., 2024) can mediate mitochondrial dynamics; the PINK1-Parkin signaling pathway and p38 MAPK pathway (Peng et al., 2025) can mediate mitochondrial autophagy. Consequently, these pathways have the potential to mitigate fibrosis resulting from oxidative stress and inflammatory responses triggered by various factors.

#### 3.3.4 How does mitochondrial quality control in alveolar type II epithelial cells participate in regulating oxidative stress, inflammation, and fibrosis?

**FIGURE 5:**
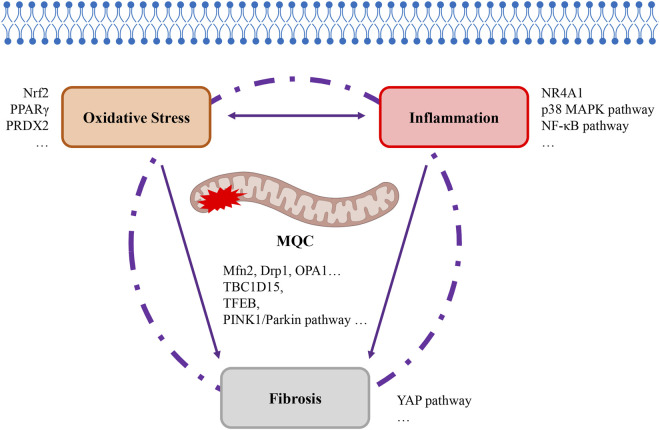
Mitochondrial quality control as a bridge linking oxidative stress, inflammation, and fibrosis. The MQC programme, which regulates mitochondrial quality within cells, has been demonstrated to be intrinsically linked to the development of oxidative stress, inflammation, and fibrosis. The interplay between oxidative stress and inflammation is a multifaceted process, with these two factors ultimately combining to precipitate the onset of fibrosis. MQC, itochondrial quality control. Drp1, dynamin-related protein 1, MAPK, mitogen-activated protein kinase, Mfn2, mitofusin 2, NF-κB, nuclear factor kappa-B, NR4A1, nuclear receptor subfamily 4 group a member 1, Nrf2, nuclear factor erythroid 2-related factor 2, OPA1, optic atrophy 1, PINK1, PTEN-induced putative kinase 1, PPAR-γ, peroxisome proliferator-activated receptor-γ, PRDX2, peroxiredoxin-2, TBC1D15, TBC domain family member 15, TFEB, transcription factor EB, YAP, yes-associated protein.

An imbalance in MQC results in the excessive production of ROS, ultimately triggering oxidative stress damage. The increased presence of cellular reactive oxygen species has been demonstrated to promote inflammatory responses by activating NF-κB (Zhu et al., 2024). This is followed by the accumulation of a large number of immune cells at the site of damage, which initiates a series of inflammatory signal transduction pathways and results in the release of a large amount of pro-inflammatory cytokines, thus resulting in a strong inflammatory response. Duan CY’s research team conducted a comprehensive analysis of the cellular landscapes in lung and blood samples from patients with severe acute respiratory syndrome caused by the novel coronavirus (SARS-CoV-2). This analysis revealed that mitochondrial dysfunction in AECII constitutes a key precipitating factor for cytokine storms and excessive inflammation (Duan et al., 2022). It is the culmination of oxidative stress and inflammatory responses that ultimately promote the stress pathways leading to fibrosis in AECII, resulting in the final outcome of fibrosis. Research has demonstrated that transplantation therapy using AECII or AECII-derived exosomes has been proposed as a means to repair damage and prevent fibrosis (Feng et al., 2023), which is significantly associated with the restoration of mitochondrial function. Consequently, the regulation of MQC in AECII can function as a unifying link between these three processes (Figure 5). Extensive research has now been conducted which indicates that disruption of MQC in AECII cells represents a critical target for the development and prognosis of ALI/ARDS. Consequently, primary research efforts have centered on therapeutic approaches targeting signaling molecules or pathways that directly or indirectly regulate MQC (Tables 1, 2). Direct regulation principally involves modulating proteins associated with mitochondrial dynamics (Mfn2, Drp1, OPA1, and so on) and mitochondrial autophagy-related signaling pathways (TBC1D15, TFEB, PINK1/Parkin pathway, and so on). The principal targets of indirect regulation are antioxidant signaling molecules (Nrf2, PPARγ, PRDX2, and so on), anti-inflammatory signaling molecules/pathways (NR4A1, p38 MAPK pathway, and NF-κB pathway, and so on) and anti-fibrosis signaling molecules/pathways (YAP pathway, and so on). By intervening in MQC through the aforementioned signaling molecules/pathways, they participate in the regulatory processes linking oxidative stress, inflammation, and fibrosis. This also encompasses histone deacetylases (HDACs), such as Sirt1, Sirt3, and HDAC3, which exert non-canonical transcriptional regulation independent of deacetylase activity. These proteins influence multiple processes, including inflammation, metabolism, cell proliferation, and apoptosis. Extensive research has been conducted on the underlying molecular mechanisms, providing robust laboratory evidence that MQC regulation in AECII serves as a bridge linking oxidative stress, inflammation, and fibrosis (Figure 5). The subsequent stage entails the identification of opportunities among these numerous molecular signals to translate these fundamental experiments into clinical applications. In particular, further development of pharmaceuticals for the early stages of ALI/ARDS should be pursued based on the aforementioned fundamental research. For instance, some researchers have proposed that the early administration of inhaled β2-adrenergic agonists (Azevedo Voltarelli et al., 2021) and other cAMP-elevating agents following alveolar injury may serve as a preventive strategy against the progression of ARDS (Sriram et al., 2021). Despite the existence of numerous clinical studies on ALI/ARDS, the precision of target identification remains significantly inadequate.

## 4 Targeted therapy for mitochondrial quality control

Despite considerable advancement in fundamental research concerning the regulation of MQC in AECII, which is advantageous for the management of oxidative stress, inflammation, and fibrosis (Tables 1, 2), there has been no corresponding progress in clinical practice. The majority of patients with ALI/ARDS continue to receive palliative treatment (Pokharel et al., 2024). The present study aims to assess the current progress in the development of targeted therapies for ALI/ARDS using MQC. To this end, a summary of relevant clinical trial reports that have been tested or are currently being tested in clinical settings has been compiled in Table 3. The research focuses on glucocorticoid receptor agonists, adrenergic receptor agonists (including α-2A, α-2B and β2 adrenergic receptors), thyroid hormone receptor agonists, low-density lipoprotein receptor-related protein 1 agonists, adrenergic receptor antagonists (β1 adrenergic receptors), aldosterone receptor antagonists, angiotensin II receptor blockers, hypoxia-inducible factor prolyl hydroxylase inhibitors (HIF-PHIs), PPARα agonists, microtubule disruptors, MAPK signaling pathway modulators, extracellular nicotinamide phosphoribosyltransferase (eNAMPT) antagonists, and anti-fibrotic drugs. Whilst the results of these studies are not consistent, there are some positive clinical outcomes reported. The primary effective studies have centered on glucocorticoid receptor agonists (NCT00562835, NCT01284452, and NCT01731795), angiotensin II receptor blockers (NCT04355936), HIF-PH inhibitors (NCT04478071), and microtubule disruptors (NCT04842747). The primary studies that were ineffective or terminated primarily involved angiotensin II receptor blockers (NCT04312009, NCT04606563) and LDL receptor-related protein 1 agonists (NCT05135624). It is also worthy of note that there are ongoing clinical trials with results yet to be published, including a selective peroxisome proliferator-activated receptor alpha (PPARα) agonist (NCT04661930) and an aldosterone receptor antagonist (NCT04977960). A significant number of studies are currently recruiting participants or in the preparatory stages, with a focus on glucocorticoid receptor agonists (NCT04545242), thyroid hormone receptor agonists (NCT04115514, NCT04725110), β2-adrenergic receptor agonists (NCT05527704), α2A-adrenergic receptor and α2B adrenergic receptor agonists (NCT05241067), β-1 adrenergic receptor blockers (NCT06013319, NCT05847517), eNAMPT antagonists (NCT05938036), MAPK signaling pathway modulators (NCT05795465), and anti-fibrotic drugs (NCT05075161). The inconsistencies observed in the aforementioned studies, particularly the rather contradictory findings regarding angiotensin II receptor blockers and β2-adrenergic receptor agonists, may be attributable to a number of factors, including the iterative nature of drug innovation and the varying degrees of patient condition specificity. Moreover, the table provides substantiating literature for the MQC that may be implicated (Azevedo Voltarelli et al., 2021; Choi and Han, 2021; Sinha et al., 2015; Helfenberger et al., 2019; Huang et al., 2022; Nakashima et al., 2024; Woods et al., 2016; Chinnarasu et al., 2021; Kurita et al., 2017; Yuan et al., 2018; Wang et al., 2015; Gui et al., 2020; Song et al., 2021; Lin et al., 2023; Zhang et al., 2024). The present paper draws upon the findings of two studies that employed multi-omics comparative analyses of urine and plasma samples from patients with ARDS induced by COVD-19 and bacterial sepsis (Batra et al., 2022; Batra et al., 2023). The results of these studies indicated that mitochondrial dysfunction plays a significant role in the development and prognosis of ARDS. In addition, it has been demonstrated to exhibit close interconnections with cell adhesion/extracellular matrix molecules and inflammation. This emphasizes the necessity for effective regulation of MQC in the prevention and treatment of ALI/ARDS, thus necessitating further investigation. Beyond the realm of drug development, clinical studies involving inhaled gases (carbon monoxide) (NCT03799874) have demonstrated a reduction in circulating mitochondrial DNA levels in the inhaled CO treatment group (Fergie et al., 2019). Mesenchymal stem cells (MSCs) have also been employed for the treatment of ARDS, with multiple clinical trials (NCT01902082, NCT01775774, NCT02097641) having validated the safety of MSC use and demonstrated improvements in ARDS-related inflammation (Zheng et al., 2014) and respiratory dysfunction (Matthay et al., 2019b) to a certain extent. Nevertheless, it is imperative to exercise caution with regard to dosage and cell viability. Research teams have discovered that mitochondrial dysfunction diminishes the therapeutic efficacy of MSCs in repairing epithelial wounds within inflammatory environments (Fergie et al., 2019). The present studies provide direct evidence that links mitochondrial dysfunction in AECs to the prognosis of ARDS. Moreover, the regulation of oxidative stress, inflammation, and fibrosis in ALI/ARDS via the MQC pathway presents transformative opportunities for the treatment of sepsis. Emerging evidence highlights the critical role of mitochondrial dynamics and mitochondrial autophagy in AECII within ALI/ARDS, suggesting that multi-targeted strategies may be required to disrupt their synergistic pathophysiology. Furthermore, innovative delivery methodologies (e.g., inhalation) could be developed to more accurately regulate MQC processes within AECII. In order to translate these insights, it is necessary to incorporate a more mechanistic approach with the biology of sepsis, in particular by means of systems approaches such as multi-omics analysis, with a view to characterizing the regulation of dynamic MQC pathways in subpopulations of patients. The potential of biomarkers, such as mtDNA, to facilitate real-time stratification for the purpose of guiding therapeutic interventions, is a promising area of research. The implementation of these techniques would facilitate improved timing of drug administration. Concurrently, a plethora of signaling molecules/pathway therapies exerting either direct or indirect influence on the MQC pathway were investigated, including Mdivi-1 (Drp1 inhibitor) (Liu et al., 2023), BGP-15 (OPA1 activator) (Liu et al., 2023), leflunomide (Mfn2 promoter) (Li C. et al., 2023), PINK1/Parkin pathway modulators (Zhu et al., 2024; Wang et al., 2024; Meng et al., 2025), Nrf2 activators (Qian et al., 2025), and activation of the PI3K/Akt pathway (Shi et al., 2019). These agents have exhibited preclinical efficacy. However, these require optimization through high-throughput screening to establish favorable safety profiles. The translation of clinical findings into practice will be contingent on the development of adaptive trial designs capable of aligning these bespoke interventions with the evolving sepsis phenotypes. The utilization of computational modelling may facilitate the prediction of individual patient responses. Methodological advances must address critical gaps, including the development of rapid MQC biomarker assays and standardized preclinical models that better reflect the heterogeneity of clinical sepsis (Wang and Liu, 2023). A collaborative framework uniting basic researchers, clinicians, and trial methodologies is essential to strike the delicate balance between therapeutic efficacy and host defense preservation, ultimately linking mechanism discovery to viable clinical strategies for this complex syndrome.

**TABLE 3:** The ARDS Clinical Trials Evaluating Drugs with evidence of mitochondrial quality control.

ClinicalTrials.gov ID	Official title	Drug name	Target/Action	Possible effect on mitochondrial quality control
NCT00434993 (2007-08∼2008-11)	Prospective, Randomized, Multicenter Trial of Aerosolized Albuterol Versus Placebo in Acute Lung Injury	Albuterol Sulfate	β2-adrenergic receptor agonist	Azevedo Voltarelli et al. (2021)
NCT00562835 (2008-02∼2009-02)	Randomized, Placebo-Controlled, Double-Blind Clinical Trial to Evaluate the Safety and Efficacy of Low-Dose Glucocorticoid Infusion in Acute Respiratory Distress Syndrome (ARDS)	Methylprednisolone	Glucocorticoid receptor agonist	Choi and Han (2021)
NCT01284452 (2010-12∼2015-03)	Efficacy of Moderate Dose Hydrocortisone in Treatment of Severe Sepsis and Septic Shock Patients With Acute Lung Injury/Acute Respiratory Distress Syndrome: A Randomized Controlled Trial	Hydrocortisone	Glucocorticoid receptor agonist	Choi and Han (2021)
NCT01731795 (2013-03-28∼2019-02-12)	A Comparative, Randomised Controlled Trial for Evaluating the Efficacy of Dexamethasone in the Treatment of Patients With Acute Respiratory Distress Syndrome	Dexamethasone	Glucocorticoid receptor agonist	Choi and Han (2021)
NCT04115514 (2019-10-21∼2025-10-31)	PHASE II RANDOMIZED, INTERVENTION VERSUS NON- INTERVENTION, MULTI- CENTER STUDY OF THE EFFECTS OF THYROID HORMONE (T3) ON SAFETY/TOLERABILITY AND OXYGENATION IN SUBJECTS WITH ACUTE RESPIRATORY DISTRESS SYNDROME (ARDS)	Liothyronine Sodium	Thyroid hormone receptors agonist	Sinha et al. (2015)
NCT04312009 (2020-04-13∼2021-02-01)	Randomized Controlled Trial of Losartan for Patients With COVID-19 Requiring Hospitalization	Losartan	Angiotensin II receptor blocker	Helfenberger et al. (2019)
NCT04355936 (2020-05-19∼2020-11-30)	Telmisartan for Treatment of COVID-19 Patients: an Open Label Randomized Trial	Telmisartan	Angiotensin II receptor blocker	Helfenberger et al. (2019)
NCT04478071 (2020-08-22∼2022-03-25)	Vadadustat for the Prevention and Treatment of Acute Respiratory Distress Syndrome (ARDS) in Hospitalized Patients With Coronavirus Disease 2019 (COVID-19)	Vadadustat	HIF-PHs inhibitor	Huang et al. (2022)
NCT04606563 (2020-10-09∼2022-04-22)	Host Response Mediators in Coronavirus (COVID-19) Infection - Is There a Protective Effect of Losartan and Other ARBs on Outcomes of Coronavirus Infection?	Losartan, Valsartan, Azilsartan, Candesartan, Eprosartan, Irbesartan, Olmesartan, Telmisartan	Angiotensin II receptor blockers	Helfenberger et al. (2019)
NCT04661930 (2021-01-01∼2022-07-01)	A Study of a 10-days Fenofibrate Treatment, or Until Discharge From Hospital, Among COVID-19 Infected Patients Requiring Hospitalization	Fenofibrate	Selective PPARα agonist	Nakashima et al. (2024)
NCT04842747 (2021-05-18∼2022-07-06)	Phase 3, Randomized, Placebo-Controlled, Efficacy and Safety Study of VERU-111 for the Treatment of Severe Acute Respiratory Syndrome Coronavirus 2 (SARS-CoV-2) in Patients at High Risk for Acute Respiratory Distress Syndrome (ARDS)	VERU-111, Sabizabulin	Microtubule disruptor	Woods et al. (2016)
NCT04545242 (2021-07-06∼2026-12-30)	Efficacy of Higher vs. Lower Doses of Dexamethasone in Patients With Acute Hypoxemic Respiratory Failure (Including ARDS) Caused by Infections (Including COVID-19)	Dexamethasone	Glucocorticoid receptor agonist	Choi and Han (2021)
NCT05135624 (2021-12-01∼2023-06-30)	SP16 as a Therapeutic for SARS-CoV-2 Induced ARDS.	SP16	LDL receptor related protein 1 agonists	Chinnarasu et al. (2021)
NCT05527704 (2021-12-31∼2026-09-30)	a Multicentre, Double-blind, Randomized, Placebo-controlled Phase III Trial of the Inhaled β2-adrenergic Receptor Agonist Salbutamol for Transient Tachypnoea of the Newborn (the REFSAL Trial)	Salbutamol	β2-adrenergic receptor agonist	Azevedo Voltarelli et al. (2021)
NCT05075161 (2022-06-01∼2025-12)	Pirfenidone to Prevent Fibrosis in ARDS. A Randomized Controlled Trial – PIONEER.	Pirfenidone	Anti-fibrotic drug	Kurita et al. (2017)
NCT04977960 (2022-09∼2023-12)	MINECRAFT Study: MINEralcorticoid Receptor Antagonism With CanRenone As eFfective Treatment in Moderate to Severe ARDS in COVID-19, a Phase 2 Clinical Trial	Potassium Canrenoate	Aldosterone receptor antagonist	Yuan et al. (2018)
NCT06013319 (2023-02-20∼2026-10-30)	Effects of Esmolol on Oxygenation Index by Controlling Heart Rate in Patients With Acute Respiratory Distress Syndrome	Esmolol	Beta-1 adrenergic receptor blocker	Wang et al. (2015)
NCT05795465 (2023-04-04∼2025-12)	A Phase 2, Two-Part Study to Evaluate the Safety and Tolerability of GEn-1124 in Subjects with Acute Respiratory Distress Syndrome (ARDS)	GEn-1124	MAPKAPK2 modulators (MAP kinase-activated protein kinase 2 modulators)、 p38α modulators (P38 α mitogen-activated protein kinase modulators)	Gui et al. (2020)
NCT05938036 (2023-12-01∼2025-08-31)	PUERTA: A P2A Multi-center, Randomized, Double-blind, Placebo-controlled Study Assessing Safety and Efficacy of the eNAMPT Targeting mAb ALT-100 in Moderate/Severe ARDS/VILI Patients	ALT-100	eNAMPT antagonist	Song et al. (2021)
NCT05847517 (2024-08-13∼2027-12)	Randomised, Double-blind, Placebo-controlled Clinical Trial to Evaluate the to Assess the Efficacy of Intravenous Metoprolol in Patients With Acute Respiratory Distress Syndrome (ARDS)	Metoprolol	Beta-1 adrenergic receptor blocker	Wang et al. (2015)
NCT05241067 (2025-08-31∼2025-12-31)	A Multicentric Randomized, Double-blind, Placebo-controlled Study to Assess the Safety and Efficacy of Centhaquine as an Adjuvant to the Standard of Care in COVID-19 Patients With Moderate to Severe Acute Respiratory Distress Syndrome	Centhaquine	alpha-2A-adrenergic receptor and Alpha-2B adrenergic receptor agonist	Lin et al. (2023), Zhang et al. (2024)
NCT04725110 (2026-01-15∼2031-10-15)	Phase II Trial of Direct Topical Lung T3 Treatment to Improve Outcome and Sequelae of COVID-19 ARDS - A Multi-Site, Randomized, Double-blinded, Placebo-Controlled Clinical Trial	T3	Thyroid hormone receptors agonist	Sinha et al. (2015)

## 5 Conclusion

The present study focuses on the crucial role of MQC regulation in AECII in mediating oxidative stress, inflammation, and subsequent fibrosis in ALI/ARDS. However, based on the current state of research, it is possible to influence the regulation of MQC in AECII by modulating key signaling molecules (e.g., Nrf2, Sirt1, etc.) and key signaling pathways (e.g., NF-κB, PINK1-Parkin, and MAPK pathways, etc.), thereby exerting corresponding antioxidant, anti-inflammatory, and anti-fibrotic effects. In addition, favorable clinical trial results provide further substantiation for the efficacy of the treatment. This is an attractive clinical target. However, due to an incomplete understanding of the cellular processes involved in regulating MQC during disease progression, the development of its therapeutic potential is currently limited. Current clinical trials also exert an indirect influence on the regulation of MQC through the mediation of related signaling molecules and signaling pathways, and do not specifically target the regulation of MQC processes in AECII, highlighting the enormous potential of this direction. This highlights the immense potential of this research direction. Advances in medicine, alongside further basic biological research and the development of new drugs, have rendered MQC in AECII a promising candidate for the prevention and treatment of ALI/ARDS.

## Glossary

AAV-Tβ4: adeno-associated virus-Tβ4
AECI: alveolar type I epithelial cells
AECII: alveolar type II epithelial cells
AECs: alveolar epithelial cells
ALI: acute lung injury
AMs: alveolar macrophages
AMP: adenosine 5′-monophosphate
AMPK: AMP-activated protein kinase
ARDS: acute respiratory distress syndrome
ARE: antioxidant response element
ATP: adenosine triphosphate
BACs: bronchoalveolar cells
BALF: bronchoalveolar lavage fluid
BLM: bleomycin
BNIP3: BCL2 interacting protein 3
**Ca** ^**2+**^: calcium ions
CL: lipid cardiolipin
cPLA2: cytoplasmic phospholipase A2
CSE: cigarette smoke extract
Cx43: connexin 43
DAMPs: damage-associated molecular patterns
Drp1: dynamin-related protein 1
EMT: epithelial-mesenchymal transition
eNAMPT: extracellular nicotinamide phosphoribosyltransferase
Fis1: fission 1
FUNDC1: FUN14 domain containing 1
gadd7: growth-arrested DNA damage-inducible gene 7
GM-CSF: granulocyte-macrophage colony-stimulating factor
GSH-Px: glutathione peroxidase
GTPases: guanosine triphosphate hydrolases
HDACs: histone deacetylases
HDAC3: histone deacetylase 3
HO-1: heme oxygenase-1
HSPB8: heat shock protein B8
IL: interleukin
IMM: inner mitochondrial membranes
IPF: idiopathic pulmonary fibrosis
KD: knockdown
KEAP1: Kelch-like ECH-associated protein 1
LC3: light chain 3
lncRNA: long non-coding RNA
L-OPA1: long OPA1
LSD1: lysine-specific demethylase 1
MAPK: mitogen-activated protein kinase
Mdivi-1: mitochondrial division inhibitor-1
MERCs: mitochondria-endoplasmic reticulum contact sites
Mff: mitochondrial fission factor
Mfn1: mitofusin 1
MiD49: mitochondrial dynamics proteins of 49 kDa
MiD51: mitochondrial dynamics proteins of 51 kDa
MLE: murine lung epithelial
MSCs: mesenchymal stem cells
mtDNA: mitochondrial DNA
mtROS: mitochondrial reactive oxygen species
MyD88: myeloid differentiation primary response protein 88
NDP52: nuclear dot protein 52
NEAT1: nuclear enriched abundant transcript 1
NF-κB: nuclear factor kappa-B
NIX: NIP3-like protein X
NLRP3: NOD-like receptor protein 3
NR4A1: nuclear receptor subfamily 4 group a member 1
Nrf2: nuclear factor erythroid 2-related factor 2
OE: overexpression
OMM: outer mitochondrial membranes
OPA1: optic atrophy 1
OPTN: optic nerve phosphodiesterase
OXPHOS: oxidative phosphorylation
PD-L: programmed death-ligand
PF: pulmonary fibrosis
PGE2: prostaglandin E2
PINK1: PTEN-induced putative kinase 1
PQ: paraquat
Prdx3: Peroxiredoxin 3
PGC-1α: PPARγcoactivator 1α
PPAR-γ: peroxisome proliferator-activated receptor-γ
PRDX2: peroxiredoxin-2
PTPN21: non-receptor protein tyrosine phosphatase 21
RHOA: ras homolog gene family
ROS: reactive oxygen species, member A
Sirt: Sirtuins
SOD: superoxide dismutase
S-OPA1: short OPA1
Stat2: signal transducers and activators of transcription 2
Tβ4: Thymosin β4
TBC1D15: TBC domain family member 15
TFEB: transcription factor EB
TFF2: trefoil factor 2
TGF: transforming growth factor
TLRs: Toll-like receptors
TOM: translocase of the outer mitochondrial membrane
ULK1: UNC-51-like kinase 1
YAP: yes-associated protein
